# The independent prognostic effect of marital status on non-small cell lung cancer patients: a population-based study

**DOI:** 10.3389/fmed.2023.1136877

**Published:** 2023-06-01

**Authors:** Dechang Zhao, Rusi Zhang, Longjun Yang, Zirui Huang, Yongbin Lin, Yingsheng Wen, Gongming Wang, Guangran Guo, Lanjun Zhang

**Affiliations:** ^1^State Key Laboratory of Oncology in South China, Collaborative Innovation Center for Cancer Medicine, Guangzhou, China; ^2^Department of Thoracic Surgery, Sun Yat-sen University Cancer Center, Guangzhou, China

**Keywords:** non-small cell lung cancer, SEER database, prognostic factors, propensity score matching, cox proportional hazard regressions

## Abstract

**Background:**

Previous studies had demonstrated that marital status was an independent prognostic factor in multiple cancers. However, the impact of marital status on non-small cell lung cancer (NSCLC) patients was still highly controversial.

**Method:**

All NSCLC patients diagnosed between 2010–2016 were selected from the Surveillance, Epidemiology and End Results (SEER) database. To control the confounding effect of related clinicopathological characteristics, propensity score matching (PSM) was conducted between married and unmarried groups. In addition, independent prognostic clinicopathological factors were evaluated via Cox proportional hazard regression. Moreover, nomograms were established based on the clinicopathological characteristics, and the predictive accuracy was assessed by calibration curves. Furthermore, decision curve analysis (DCA) was used to determine the clinical benefits.

**Results:**

In total, 58,424 NSCLC patients were enrolled according to the selection criteria. After PSM, 20,148 patients were selected into each group for further analysis. The married group consistently demonstrated significantly better OS and CSS compared to unmarried group [OS median survival (95% CI): 25 (24–26) vs. 22 (21–23) months, *p* < 0.001; CSS median survival (95% CI): 31 (30–32) vs. 27 (26–28) months, *p* < 0.001]. Moreover, single patients were associated with the worst OS [median survival (95% CI): 20 (19–22) months] and CSS [median survival (95%CI): 24 (23–25) months] among unmarried subgroups. Besides, unmarried patients had a significantly worse prognosis compared to married patients in both univariate and multivariate Cox proportional hazard regressions. Furthermore, married group was associated with better survival in most subgroups. To predict the 1-, 3- and 5-year OS and CSS probabilities, nomograms were established based on age, race, sex, gender, marital status, histology, grade, TNM stage. The C-index for OS and CSS were 0.759 and 0.779. And the calibration curves showed significant agreement between predictive risk and the observed probability. DCA indicated nomograms had consistently better predict performance.

**Conclusion:**

This study demonstrated that unmarried NSCLC patients were associated with significantly worse OS and CSS compared to married NSCLC patients. Therefore, unmarried patients need not only closer surveillance, but also more social and family support, which may improve patients’ adherence and compliance, and eventually improve the survival.

## Introduction

Lung cancer is one of the most prevalent and deadly cancers in the world and causes more deaths than breast, prostate, colorectal, and brain cancers combined (1). Non-small cell lung cancer (NSCLC) is a major histological type of lung cancer and accounts for approximately 85% of all lung cancer cases (2). Currently, the AJCC TNM stage, which focuses on the anatomic extent of the tumour, is the most important prognostic factor for NSCLC (3). Moreover, many prognostic factors, such as age, gender, histology and ethnicity, have been demonstrated in previous studies (3, 4).

The divorce rate has been increasing for the last 40 years, especially in the last 20 years. And more and more people marry at a later age or even do not marry at all. According to the study between 1960 and 2010, divorce got more and more common, and people started marrying later than before or not marrying at all (5). Especially during 1990–2010, the divorce rate has doubled among the elderly, which attracted more and more researchers’ attention (6). Furthermore, marital status has been found to impact the physical condition of patients by influencing the adherence to treatments and the level of economic resources (7, 8). In recent years, an increasing number of studies have demonstrated that marital status is an independent prognostic factor in several cancers, such as breast cancer (9, 10), gastric adenocarcinoma (11), pancreatic cancer (12), and primary liver cancer (13). However, studies from Jatoi et al., Siddiqui et al. and Saito-Nakaya et al. demonstrated that marital status was not prognostic of NSCLC survival (14–16).

The Surveillance, Epidemiology and End Results (SEER) cancer database, which is funded by the National Cancer Institute, provides demographic information and cancer characteristics of several cancers. Approximately 28% of the population in the USA is covered in the SEER database (17). In this study, we aimed to perform a retrospective study on a large population to identify the prognostic influence of marital status on the survival of patients diagnosed with NSCLC. Meanwhile, propensity score matching methods were used to control confounding factors.

## Method

### Patient selection

All NSCLC patients diagnosed between 2010–2016 were selected from the SEER database. The specific inclusion criteria were as follows: (1) patients older than 17 years; (2) patients diagnosed between 2010–2016; (3) patients diagnosed with only one primary lung cancer (ICD-O-3 primary site codes: C340-343 and C348-349); (4) patients diagnosed with NSCLC (ICD-O-3 histology code: large cell carcinoma 8012, 8013, and 8014; adenocarcinoma 8140–8147, 8250–8255, 8310, 8333, 8470, 8480, 8481, 8490, 8550, and 8551; squamous cell carcinoma 8052, 8070-8078 and 8083; and adenosquamous cell carcinoma 8560).

The exclusion criteria were as follows: (1) patients diagnosed with other primary cancers; (2) patients without complete demographic and cancer characteristic information; (3) patients without positive histological or immunophenotyping diagnosis; (4) patients without complete information about therapy; (5) patients without complete survival states and time; and (6) patients with a time of follow-up less than 1 month. And the flow chart for patient selection was shown in Supplementary Figure S1.

Finally, 58,424 patients were selected for our study based on the above selection criteria, and related clinicopathological characteristics including age, gender, race, marital status, histology, grade, TNM stage, therapy, survival state, and survival time were extracted. In addition, November 2019 was set as the last follow-up, and overall survival (OS) and cancer-specific survival (CSS) as the primary outcomes were separately defined as the follow-up between diagnosis and death or cancer-specific death.

According to the regulation of the SEER database, we obtained permission to access the research data, and the reference number was 14,683-Nov2019. Since the SEER database is publicly available and all patients were deidentified, ethical approval was waivered by the ethical committee of our hospital.

### Propensity score matching

The marital status of all the patients was dichotomized as married and unmarried, and the latter was further specified into single, separated, divorced, and widowed statuses. To accurately identify the difference of marital status in NSCLC patients, 1:1 propensity score matching (PSM) was conducted to control confounding clinicopathological characteristics between the two groups by using the nearest-neighbour algorithm with a calliper of 0.0001, including age, gender, race, histology, grade, TNM stage and therapy.

### Statistics analysis

In our study, the National Cancer Institute’s SEER*Stat software [version 8.3.6; SEER 18 Regs Custom Data (with additional treatment fields), November 2019 Sub (1975–2016 varying) database] was used in this study. All related clinicopathological characteristics were presented with descriptive statistics, including number and percentage. Pearson’s chi-square tests were used to examine the difference between marital status and other variables. The survival curves of OS and CSS were performed by using the Kaplan–Meier method, and the difference between survival curves was assessed by using log-rank tests. Additionally, we assessed the hazard ratios (HRs) and 95% confidence intervals (CIs) of all the variables of the PS-matched cohort over OS and CSS via univariable and multivariable Cox proportional hazards regression models with the Enter method.

To better predict the prognosis of NSCLC patients, the nomograms were constructed by clinicopathological characteristics, including age, race, sex, gender, marital status, histology, grade, TNM stage. And 1-, 3- and 5-year OS and CSS probabilities were estimated using the nomogram. Concordance index (C-index) was used to evaluate discriminative ability, and Calibration plots to evaluate calibrating ability. Typically, C-index values greater than 0.7 suggested a reasonable estimation. In addition, decision curve analysis (DCA) were used to evaluate the clinical benefits and utility of the nomogram compared with T, N, M stage.

All statistical tests were two-sided, and a value of *p* < 0.05 was regarded as a statistically significant difference. Statistical analyses were performed using Statistical Product and Service Solutions (SPSS version 26.0; IBM Corporation, Armonk, NY, United States) and R (version 3.6.3; R Development Core Team, http://www.r-project.org).

## Results

### Clinicopathological characteristics of patients

A total of 58,424 patients were enrolled in this study, among which 32,025 (54.8%) were married and 26,399 (45.2%) were unmarried. Detailed demographic and clinicopathological characteristics are shown in Table 1, and these characteristics are stratified by marital status. Chi-square tests demonstrated significant differences in most of the other variables between different marital status, including gender (*p* < 0.001), race (*p* < 0.001), histology (*p* < 0.001), grade (*p* < 0.001), stage (*p* = 0.016), T stage (*p* < 0.001), M stage (*p* = 0.005), surgery of primary site (*p* < 0.001), intraoperative lymph node evaluation (*p* < 0.001), and chemotherapy (*p* < 0.001). Additionally, clinicopathological characteristics comparison by the single, separated, divorced and widowed patients within unmarried group were shown in Supplementary Table S1.

**Table 1:** Clinicopathological characteristics of NSCLC patients in SEER database before propensity score matching.

Variable	Total	Married	Unmarried	*p*-value
	(*n* = 58,424)	(n = 32,025, 54.8%)	(*n* = 26,399, 45.2%)	
Gender				*p* < 0.001
Male	30,296 (51.9%)	19,319 (60.3%)	10,977 (41.6%)	
Female	28,128 (48.1%)	12,706 (39.7%)	15,422 (58.4%)	
Age				*p* = 0.386
≤65	23,146 (39.6%)	12,636 (39.5%)	10,510 (39.8%)	
>65	35,278 (60.4%)	19,389 (60.5%)	15,889 (60.2%)	
Race				*p* < 0.001
White	47,010 (80.5%)	26,470 (82.7%)	20,540 (77.8)	
Black	6,710 (11.5%)	2,372 (7.4%)	4,338 (16.4%)	
Other	4,707 (8.1%)	3,183 (9.9%)	1,521 (5.8%)	
Histology				*p* < 0.001
ADC	35,374 (60.5%)	19,863 (62.0%)	15,511 (58.8%)	
SCC	20,585 (35.2%)	10,821 (33.8%)	9,764 (37.0%)	
LCC	1,215 (2.1%)	628 (2.0%)	587 (2.2%)	
ASC	1,250 (2.1%)	713 (2.2%)	537 (2.0%)	
Grade				*p* < 0.001
Well differentiated	6,572 (11.2%)	3,770 (11.8%)	2,802 (10.6%)	
Moderately differentiated	23,324 (39.9%)	12,918 (40.3%)	10,406 (39.4%)	
Poorly differentiated	27,581 (47.2%)	14,849 (46.4%)	12,732 (48.2%)	
Undifferentiated	947 (1.6%)	488 (1.5%)	459 (1.7%)	
Stage				*p* = 0.016
I	19,051 (32.6%)	10,586 (33.0%)	8,483 (32.1%)	
II	7,817 (13.4%)	4,339 (13.5%)	3,478 (13.2%)	
III	12,899 (22.1%)	7,047 (22.0%)	5,852 (22.2%)	
IV	18,657 (31.9%)	10,071 (31.4%)	8,586 (32.5%)	
T Stage				*p* < 0.001
1	15,658 (26.8%)	8,720 (27.2%)	6,938 (26.3%)	
2	19,725 (33.8%)	10,965 (34.2%)	8,760 (33.2%)	
3	11,857 (20.3%)	6,473 (20.2%)	5,384 (20.4%)	
4	11,184 (19.1%)	5,867 (18.3%)	5,317 (20.1%)	
N Stage				*p* = 0.117
0	30,105 (51.5%)	16,505 (51.5%)	13,600 (51.5%)	
1	6,117 (10.5%)	3,414 (10.7%)	2,703 (10.2%)	
2	16,927 (29.0%)	9,178 (28.7%)	7,749 (29.4%)	
3	5,275 (9.0%)	2,928 (9.1%)	2,347 (8.9%)	
M stage				*p* = 0.005
0	39,767 (68.1%)	21,954 (68.6%)	17,813 (67.5%)	
1	18,657 (31.9%)	10,071 (31.4%)	8,586 (32.5%)	
Surgery of primary site				*p* < 0.001
No	31,304 (53.6%)	16,096 (50.3%)	15,208 (57.6%)	
Yes	27,120 (46.4%)	15,929 (49.7%)	11,191 (42.4%)	
Intraoperative lymph node evaluation				*p* < 0.001
No	30,865 (52.8%)	15,809 (49.4%)	15,056 (57.0%)	
Yes	27,559 (47.2%)	16,216 (50.6%)	11,343 (43.0%)	
Chemotherapy				*p* < 0.001
No	32,318 (55.3%)	16,696 (52.1%)	15,622 (59.2%)	
Yes	26,106 (44.7%)	15,329 (47.9%)	10,777 (40.8%)	
Radiotherapy				*p* = 0.198
No	35,966 (61.6%)	19,790 (61.8%)	16,176 (61.3%)	
Yes	22,458 (38.4%)	12,235 (38.2%)	10,223 (38.7%)	

ADC, adenocarcinoma; SCC, squamous cell carcinoma; LCC, large cell carcinoma; ASC, adenosquamous carcinoma.

### Survival analysis

**Figure 1:**
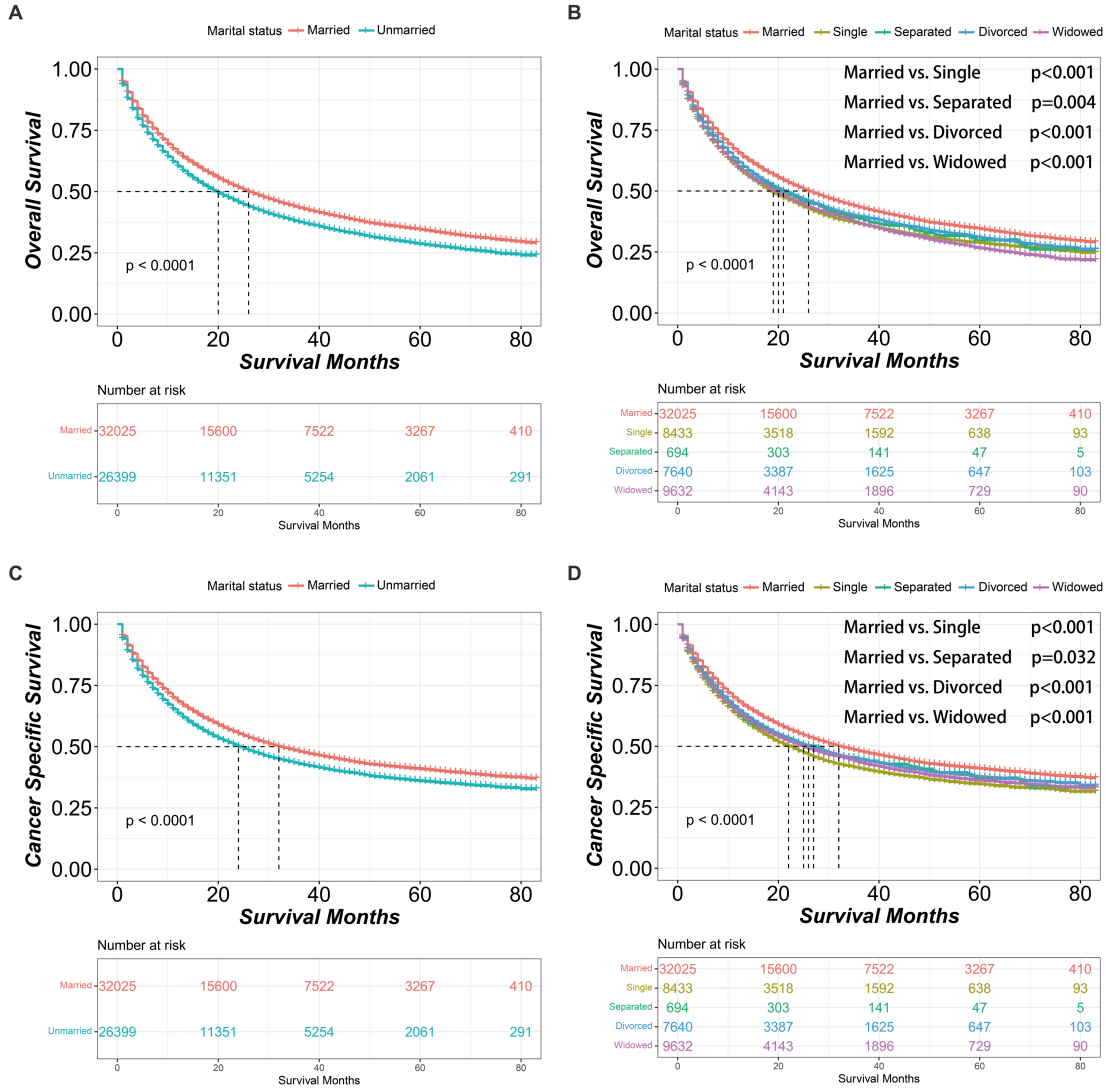
Kaplan–Meier survival curves of non-small cell lung cancer patients according to marital status before propensity score matching. **(A)** Overall survival comparison between married and unmarried patients. **(B)** Overall survival comparison among married, single, separated, divorced and widowed patients. **(C)** Cancer specific survival comparison between married and unmarried patients. **(D)** Cancer specific survival comparison among married, single, separated, divorced and widowed patients. The color band represents the 95% confidence interval of survival curves.

Before PSM, the married group had significantly better OS and CSS outcomes than the unmarried group [OS median survival (95% CI): 26 (26–27) vs. 20 (19–20) months, *p* < 0.001; CSS median survival (95% CI): 32 (31–34) vs. 24 (24–25) months, *p* < 0.001]. Within the unmarried group, patients who were divorced had better OS [median survival (95% CI): 21 (20–23) months] compared to those who were single [median survival (95% CI): 19 (18–20) months, *p* < 0.001] or widowed [median survival (95% CI): 20 (19–20) months, *p* < 0.001]. In addition, single patients had worse CSS [median survival (95% CI): 22 (21–24) months] than divorced patients [median survival (95% CI): 26 (25–28) months, *p* < 0.001] or widowed patients [median survival (95% CI): 25 (24–26) months, *p* = 0.016] (Figure 1).

Since most clinicopathological characteristics between married and unmarried groups were significantly different, PSM was performed to balance potentially confounding factors. The married group was matched at a 1:1 ratio with the unmarried group. After matching, 20,148 pairs were selected into further analysis, and all clinicopathological characteristics between the married and unmarried groups were well balanced without significant difference, as examined by chi-square tests (Table 2; Supplementary Figure S2).

**Table 2:** Clinicopathological characteristics of NSCLC patients in SEER database after propensity score matching.

Variable	Total	Married	Unmarried	*p*-value
	(n = 40,296)	(n = 20,148, 50.0%)	(n = 20,148, 50%)	
Gender				*p* = 0.952
Male	19,210 (47.7%)	9,608 (47.7%)	9,602 (47.7%)	
Female	21,086 (52.3%)	10,540 (52.3%)	10,546 (52.3%)	
Age				*p* = 0.871
≤65	16,622 (41.2%)	8,303 (41.2%)	8,319 (41.3%)	
>65	40,296 (58.8%)	11,845 (58.8%)	11,829 (58.7%)	
Race				*p* = 0.721
White	33,888 (84.1%)	16,954 (84.1%)	16,934 (84.0%)	
Black	4,010 (10.0%)	1984 (9.8%)	2026 (10.1%)	
Other	2,398 (6.0%)	1,210 (6.0%)	1,188 (5.9%)	
Histology				*p* = 0.966
ADC	24,913 (61.8%)	12,444 (61.8%)	12,469 (61.9%)	
SCC	14,131 (35.1%)	7,070 (35.1%)	7,061 (35.0%)	
LCC	635 (1.6%)	320 (1.6%)	315 (1.6%)	
ASC	617 (1.5%)	314 (1.6%)	303 (1.5%)	
Grade				*p* = 0.986
Well differentiated	4,309 (10.7%)	2,143 (10.6%)	2,166 (10.8%)	
Moderately differentiated	16,234 (40.3%)	8,119 (40.3%)	8,115 (40.3%)	
Poorly differentiated	19,302 (47.9%)	9,661 (48.0%)	9,641 (47.9%)	
Undifferentiated	451 (1.1%)	225 (1.1%)	226 (1.1%)	
Stage				*p* = 0.993
I	13,348 (33.1%)	6,665 (33.1%)	6,683 (33.2%)	
II	5,191 (12.9%)	2,591 (12.9%)	2,600 (12.9%)	
III	8,569 (21.3%)	4,285 (21.3%)	4,248 (21.3%)	
IV	13,188 (32.7%)	6,607 (32.8%)	6,581 (32.7%)	
T stage				*p* = 0.709
1	11,003 (27.3%)	5,532 (27.5%)	5,471 (27.2%)	
2	13,447 (33.4%)	6,746 (33.5%)	6,701 (33.3%)	
3	8,115 (20.1%)	4,044 (20.1%)	4,071 (20.2%)	
4	7,731 (19.2%)	3,826 (19.0%)	3,905 (19.4%)	
N stage				*p* = 0.882
0	20,810 (51.6%)	10,389 (51.6%)	10,421 (51.7%)	
1	4,162 (10.3%)	2070 (10.3%)	2092 (10.4%)	
2	11,682 (29.0%)	5,847 (29.0%)	5,835 (29.0%)	
3	3,642 (9.0%)	1842 (9.1%)	1800 (8.9%)	
M stage				*p* = 0.783
0	27,108 (67.3%)	13,541 (67.2%)	13,567 (67.3%)	
1	13,188 (32.7%)	6,607 (32.8%)	6,581 (32.7%)	
Surgery of primary site				*p* = 0.811
No	21,576 (53.5%)	10,776 (53.5%)	10,800 (53.6%)	
Yes	18,720 (46.5%)	9,372 (46.5%)	9,348 (46.4%)	
Intraoperative lymph node evaluation				*p* = 0.992
No	21,309 (52.9%)	10,655 (52.9%)	10,654 (52.9%)	
Yes	18,987 (47.1%)	9,493 (47.1%)	9,494 (47.1%)	
Chemotherapy				*p* = 0.904
No	22,420 (55.6%)	11,216 (55.7%)	11,204 (55.5%)	
Yes	17,876 (44.4%)	8,932 (44.3%)	8,944 (44.4%)	
Radiotherapy				*p* = 0.821
No	25,162 (62.4%)	12,592 (62.5%)	12,570 (62.4%)	
Yes	15,134 (37.6%)	7,556 (37.5%)	7,578 (37.6%)	

ADC, adenocarcinoma; SCC, squamous cell carcinoma; LCC, large cell carcinoma; ASC, adenosquamous carcinoma.

**Figure 2:**
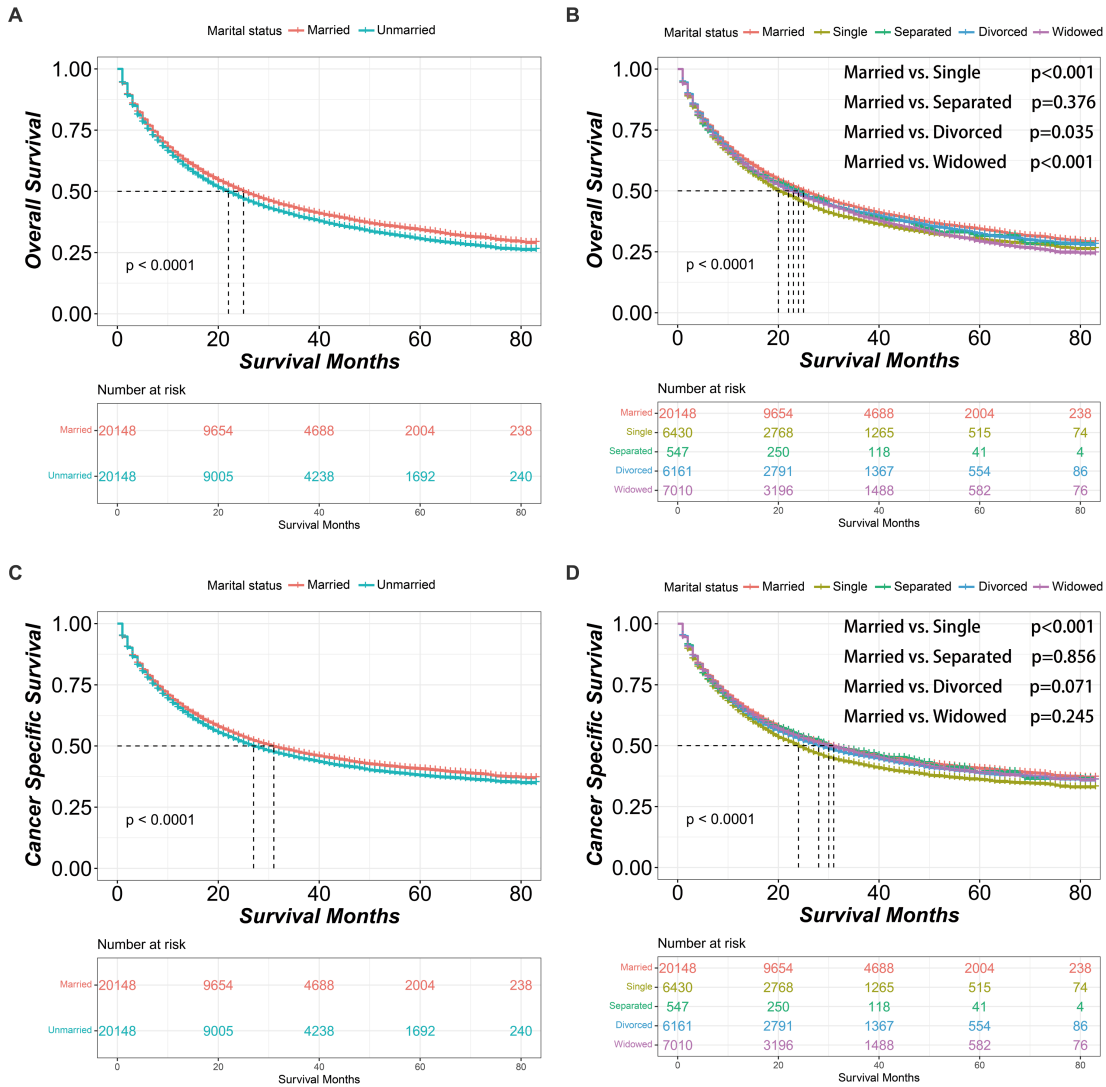
Kaplan–Meier survival curves of non-small cell lung cancer patients according to marital status after propensity score matching. **(A)** Overall survival comparison between married and unmarried patients. **(B)** Overall survival comparison among married, single, separated, divorced and widowed patients. **(C)** Cancer specific survival comparison between married and unmarried patients. **(D)** Cancer specific survival comparison among married, single, separated, divorced and widowed patients. The color band represents the 95% confidence interval of survival curves.

After PSM, the married group consistently demonstrated better OS and CSS outcomes than unmarried group [OS median survival (95% CI): 25 (24–26) vs. 22 (21–23) months, *p* < 0.001; CSS median survival (95% CI): 31 (30–32) vs. 27 (26–28) months, *p* < 0.001]. Similarly, within the unmarried group, the divorced patients had better OS [median survival (95% CI): 23 (22–24) months] than single patients [median survival (95% CI): 20 (19–22) months, *p* = 0.006]. Additionally, single patients had worse CSS [median survival (95% CI): 24 (23–25) months] than divorced patients [median survival (95% CI): 28 (26–30) months, *p* = 0.003] or widowed patients [median survival (95% CI): 30 (28–33) months, *p* < 0.001] (Figure 2). And after PSM, the clinicopathological characteristics of subgroups within unmarried patients were further shown in Supplementary Table S2.

### Cox proportional hazards regression model

To further explore whether different marital statuses had different impact on OS and CSS, we analysed all related variables, including marital status, with a Cox proportional hazards regression model. And Schoenfeld residual plots were performed to test the proportional hazards assumption (Supplementary Figure S3). In univariable analysis, single patients had significantly worse OS [HR (95% CI): 1.047 (1.009–1.087), *p* = 0.015] and CSS [HR (95% CI): 1.045 (1.004–1.087), *p* = 0.031] than married patients, and so did the divorced patients compared to married patients [OS: HR (95% CI): 1.125 (1.085–1.166), *p* < 0.001; CSS: HR (95% CI): 1.131 (1.089–1.175), *p* < 0.001]. In addition, the widowed patients were associated with worse OS compared to married patients [HR (95% CI): 1.093 (1.056–1.132), *p* < 0.001]. We also performed multivariable Cox proportional hazards regression analysis. And all subgroups but separated patients within unmarried group had significantly worse survival compared to married group [OS: single HR (95% CI): 1.089 (1.050–1.129), *p* < 0.001], separated [HR (95% CI): 1.144 (1.025–1.277), *p* = 0.017), divorced (HR (95% CI): 1.139 (1.097–1.182), *p* < 0.001] and widowed [HR (95% CI): 1.137 (1.097–1.179), *p* < 0.001]; CSS: single [HR (95% CI): 1.076 (1.035–1.119), *p* < 0.001], separated [HR (95% CI): 1.084 (0.961–1.222), *p* = 0.189], divorced [HR (95% CI): 1.131 (1.087–1.177), *p* < 0.001] and widowed [HR (95% CI): 1.097 (1.055–1.141), *p* < 0.001] (Table 3).

**Table 3:** Univariate and multivariate analysis of overall survival and cancer specific survival after propensity score matching.

Variable	Univariate				Multivariate			
	Overall survival		Cancer specific survival		Overall survival		Cancer specific survival	
	Hazard ratio (95%CI)	*p*-value	Hazard ratio (95%CI)	*p*-value	Hazard ratio (95%CI)	*p*-value	Hazard ratio (95%CI)	*p*-value
Gender								
Male	Reference		Reference		Reference		Reference	
Female	0.618 (0.602–0.634)	<0.001	0.624 (0.607–0.642)	<0.001	0.771 (0.751–0.792)	<0.001	0.788 (0.766–0.811)	<0.001
Age								
≤65	Reference		Reference		Reference		Reference	
>65	1.130 (1.101–1.160)	<0.001	1.034 (1.006–1.063)	<0.001	1.215 (1.181–1.249)	<0.001	1.159 (1.125–1.194)	<0.001
Race								
White	Reference		Reference		Reference		Reference	
Black	1.152 (1.106–1.200)	<0.001	1.170 (1.120–1.222)	<0.001	0.937 (0.899–0.977)	0.002	0.927 (0.887–0.969)	0.001
Other	0.836 (0.790–0.885)	<0.001	0.896 (0.844–0.950)	<0.001	0.744 (0.703–0.788)	<0.001	0.775 (0.730–0.823)	<0.001
Histology								
ADC	Reference		Reference		Reference		Reference	
SCC	1.480 (1.442–1.520)	<0.001	1.399 (1.360–1.439)	<0.001	1.182 (1.149–1.216)	<0.001	1.133 (1.099–1.168)	<0.001
LCC	1.651 (1.504–1.812)	<0.001	1.697 (1.540–1.870)	<0.001	1.394 (1.257–1.545)	<0.001	1.423 (1.277–1.586)	<0.001
ASC	1.114 (1.003–1.236)	0.043	1.061 (0.947–1.189)	0.307	1.177 (1.060–1.307)	0.002	1.149 (1.025–1.288)	0.017
Marital status								
Married	Reference		Reference		Reference		Reference	
Single	1.047 (1.009–1.087)	0.015	1.045 (1.004–1.087)	0.031	1.089 (1.050–1.129)	<0.001	1.076 (1.035–1.119)	<0.001
Separated	1.059 (0.949–1.182)	0.305	1.018 (0.903–1.147)	0.772	1.144 (1.025–1.277)	0.017	1.084 (0.961–1.222)	0.189
Divorced	1.125 (1.085–1.166)	<0.001	1.131 (1.089–1.175)	<0.001	1.139 (1.097–1.182)	<0.001	1.131 (1.087–1.177)	<0.001
Widowed	1.093 (1.056–1.132)	<0.001	1.028 (0.990–1.068)	0.154	1.137 (1.097–1.179)	<0.001	1.097 (1.055–1.141)	<0.001
Grade								
Well differentiated	Reference		Reference		Reference		Reference	
Moderately differentiated	1.919 (1.815–2.028)	<0.001	2.050 (1.926–2.182)	<0.001	1.380 (1.304–1.461)	<0.001	1.410 (1.324–1.503)	<0.001
Poorly differentiated	2.981 (2.824–3.147)	<0.001	3.341 (3.144–3.551)	<0.001	1.617 (1.528–1.710)	<0.001	1.675 (1.573–1.784)	<0.001
Undifferentiated	3.073 (2.719–3.472)	<0.001	3.492 (3.065–3.978)	<0.001	1.533 (1.340–1.753)	<0.001	1.577 (1.366–1.821)	<0.001
Stage								
I	Reference		Reference					
II	2.058 (1.975–2.164)	<0.001	2.736 (2.577–2.904)	<0.001				
III	3.779 (3.627–3.937)	<0.001	5.556 (5.289–5.837)	<0.001				
IV	7.952 (7.657–8.258)	<0.001	12.159 (11.611–12.732)	<0.001				
T stage								
1	Reference		Reference		Reference		Reference	
2	2.073 (1.994–2.155)	<0.001	2.475 (2.367–2.589)	<0.001	1.487 (1.428–1.549)	<0.001	1.672 (1.596–1.752)	<0.001
3	3.341 (3.208–3.480)	<0.001	4.223 (4.032–4.423)	<0.001	1.740 (1.664–1.820)	<0.001	1.989 (1.891–2.091)	<0.001
4	4.611 (4.428–4.801)	<0.001	5.982 (5.715–6.262)	<0.001	1.769 (1.691–1.852)	<0.001	2.038 (1.937–2.145)	<0.001
N stage								
0	Reference		Reference		Reference		Reference	
1	1.827 (1.749–1.909)	<0.001	2.089 (1.992–2.190)	<0.001	1.634 (1.562–1.710)	<0.001	1.752 (1.668–1.840)	<0.001
2	3.194 (3.101–3.289)	<0.001	3.822 (3.701–3.948)	<0.001	1.707 (1.649–1.768)	<0.001	1.838 (1.771–1.909)	<0.001
3	4.116 (3.949–4.289)	<0.001	5.002 (4.789–5.225)	<0.001	1.745 (1.665–1.828)	<0.001	1.875 (1.785–1.969)	<0.001
M stage								
0	Reference		Reference		Reference		Reference	
1	4.150 (4.042–4.261)	<0.001	4.854 (4.719–4.993)	<0.001	2.009 (1.945–2.075)	<0.001	2.132 (2.060–2.206)	<0.001
Surgery of primary site								
No	Reference		Reference		Reference		Reference	
Yes	0.185 (0.180–0.191)	<0.001	0.154 (0.149–0.159)	<0.001	0.432 (0.407–0.458)	<0.001	0.398 (0.374–0.424)	<0.001
Intraoperative lymph node evaluation								
No	Reference		Reference		Reference		Reference	
Yes	0.223 (0.216–0.229)	<0.001	0.195 (0.188–0.201)	<0.001	0.721 (0.685–0.758)	<0.001	0.732 (0.694–0.773)	<0.001
Chemotherapy								
No	Reference		Reference		Reference		Reference	
Yes	1.559 (1.520–1.599)	<0.001	1.780 (1.732–1.830)	<0.001	0.571 (0.554–0.589)	<0.001	0.589 (0.570–0.608)	<0.001
Radiotherapy								
No	Reference		Reference		Reference		Reference	
Yes	2.090 (2.037–2.144)	<0.001	2.209 (2.150–2.271)	<0.001	1.006 (0.977–1.036)	0.689	1.017 (0.986–1.049)	0.274

As the stage has the close relationship with T, N, M stage, we excluded the stage in multivariate analysis of OS and CSS. ADC, adenocarcinoma; SCC, squamous cell carcinoma; LCC, large cell carcinoma; ASC adenosquamous carcinoma.

**Figure 3:**
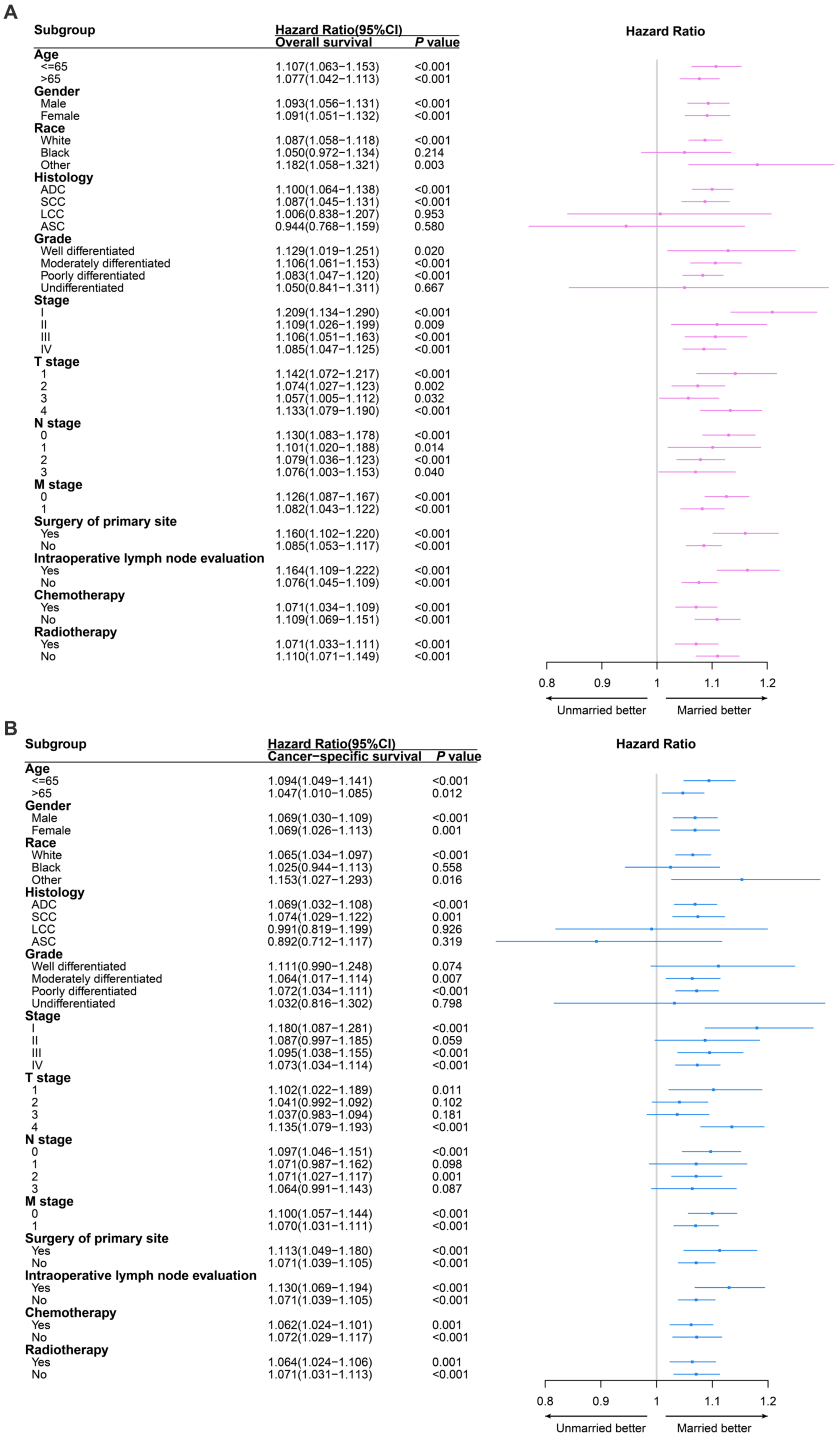
Survival comparison between married non-small cell lung cancer (NSCLC) patients and unmarried NSCLC patients in different clinicopathological subgroups analysis. Red and blue boxes represent the hazard ratios (HRs) of overall survival **(A)** and cancer specific survival **(B)** respectively. The lines represent the 95% confidence interval of hazard ratio (HR). ADC, adenocarcinoma; SCC, squamous cell carcinoma; LCC, large cell carcinoma; ASC, adenosquamous carcinoma.

In further subgroup analysis, all subgroups of age, gender, TNM stage, surgery of primary site, intraoperative lymph node evaluation, chemotherapy and radiotherapy, unmarried patients had significantly worse OS compared to married patients. Similarly, we demonstrated that unmarried patients also had significantly worse CSS in the most of subgroups compared to married patients (Figure 3).

### Construction and validation of nomogram

**Figure 4:**
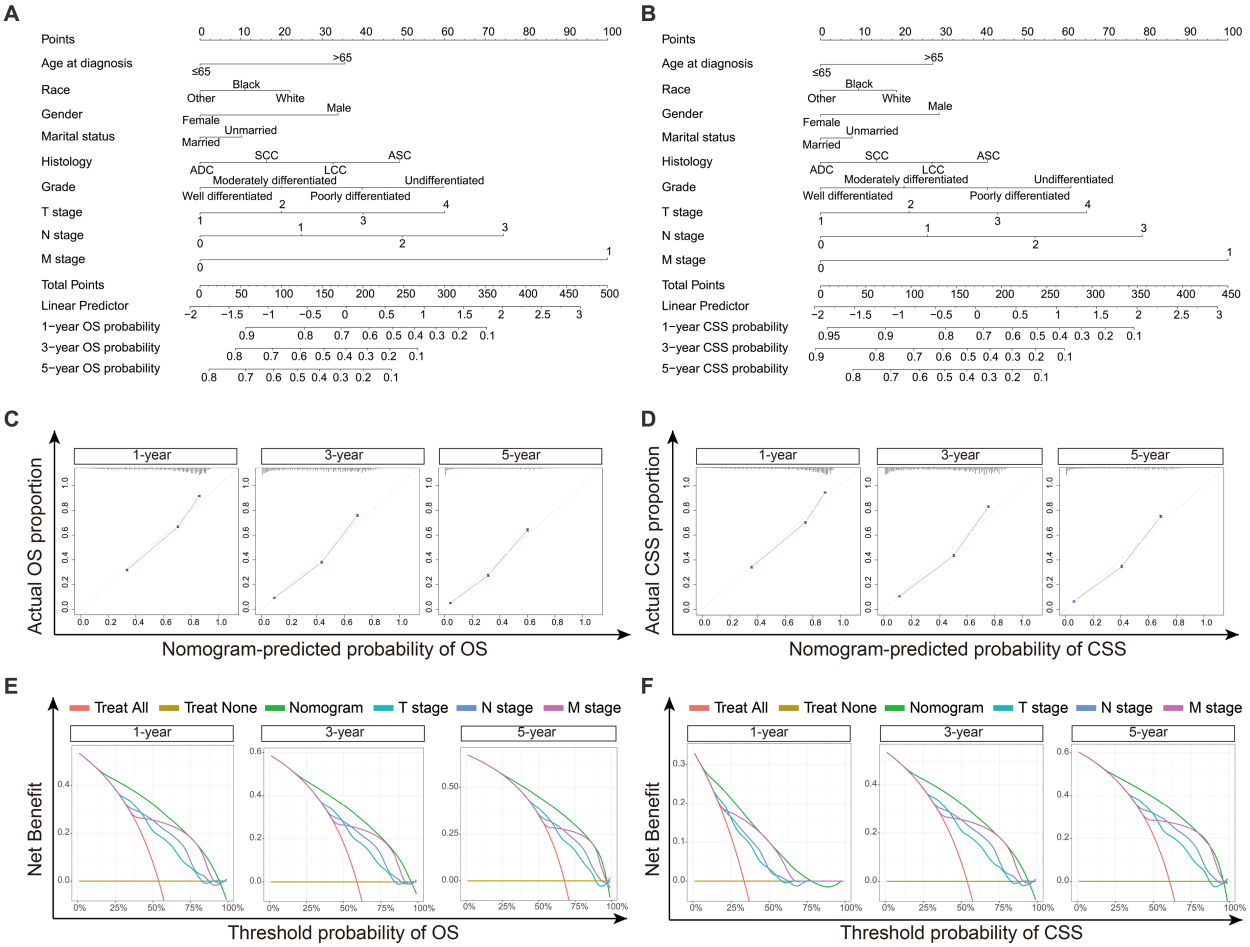
The construction and validation of Nomograms. **(A)** Nomogram model predicting the 1-, 3- and 5-year OS in NSCLC patients. **(B)** Nomogram model predicting the 1-, 3- and 5-year CSS in NSCLC patients. The nomogram is used by summing all points identified on the scale for each variable. The total points projected on the bottom scales indicate the probabilities of 1-, 3- and 5-year survival. **(C)** The calibration curves for predicting 1-, 3- and 5-year OS in NSCLC patients. **(D)** The calibration curves for predicting 1-, 3- and 5-year CSS in NSCLC patients. The OS and CSS predicted by the nomograms is plotted on the *x*-axis, and the actual OS and CSS is plotted on the *y*-axis. **(E)** Decision curve analysis (DCA) for the nomograms and TNM stage in prediction of 1-, 3- and 5-year OS in NSCLC patients. **(F)** DCA for the nomograms and TNM stage in prediction of 1-, 3- and 5-year CSS in NSCLC patients. The x-axis represents the threshold probabilities, and the *y*-axis measures the net benefit calculated by adding the true positives and subtracting the false positives. ADC, adenocarcinoma; SCC, squamous cell carcinoma; LCC large cell carcinoma; ASC, adenosquamous carcinoma.

The nomograms were constructed for predicting the OS and CSS of NSCLC patients. Specifically, each subtype of age, race, sex, gender, marital status, histology, grade, TNM stage was given a score on the point scale axis, and a total score could be calculated to estimate the 1-, 3- and 5-year OS and CSS probabilities of patients (Figures 4A,B). In addition, internal validation was utilized to test the nomograms. The calibration curves of the nomograms showed good agreement between the predictive risk and the observed probability of 1-, 3- and 5-year OS and CSS (Figures 4C,D). And the C-index values of nomograms for OS and CSS were 0.759 and 0.779, representing the reasonable estimation. Moreover, comparing the TNM stage, DCA exhibited significantly better net benefits in nomograms among 1-, 3- and 5-year OS and CSS probabilities, indicating a greater potential for clinical decision making (Figures 4E,F).

## Discussion

In the present study, we demonstrated that married patients had a significantly better prognosis than unmarried patients with a population-based database. Moreover, after adjusting for confounding clinicopathological characteristics by PSM, married group was still associated with better survival outcomes. Within unmarried patients, single patients always had a shorter median survival time in terms of OS and CSS than that in other unmarried patients. Additionally, when cofounding factors were controlled by the Cox proportional hazards regression, we still found that unmarried patients had a higher risk than married patients. In the subgroup analysis, married patients were consistently associated with better survival in most subgroups.

The conclusions of this study are consistent with previous studies conducted in other cancers (9–13) that married patients had significantly better prognosis than unmarried patients. However, few studies had investigated the impact of marital status on NSCLC survival. In a previous study by Jatoi et al. (14), with 5,898 NSCLC patients diagnosed in 1999–2006, no significant differences in survival or quality of life was found by different marital status. In addition, Siddiqui et al. (15) and Saito-Nakaya et al. (16) enrolled 1,365 and 238 NSCLC patients diagnosed during 1990s, respectively, and showed that marital status was not an independent prognostic factor. We believe our results contradicted to theirs were mainly due to the following reasons: firstly, a total of 58,424 patients were enrolled in this study, which was significantly larger sample size than previous studies; secondly, the SEER database covered as much as 28% population from the USA diagnosed between 2010 and 2016, which is more representative to the latest trend of marital status in modern society.

Multiple reasons account for the impact of marital status on NSCLC survival. Marital psychological effect is found to be one of the most important ones. Holt-Lunstad et al. (18) found that familial support from a marriage had a significant positive influence on long-term health. In contrast, loneliness and isolation associated with unmarried status may lead to unhealthy life style, including smoking and low physical activity (19). Moreover, Steptoe et al. (20) observed that married participants had lower loneliness scores than single and divorced participants, which might significantly improve their health. And a prospective study further suggested that a higher degree of optimism was associated with a lower mortality risk (21). Furthermore, psychological factors were found to have a strong impact on tumour immune microenvironment (22). The study of Reiche et al. (23) demonstrated that depression and stress decreased cytotoxic T-cell and natural killer cell activities, which might decrease the immune surveillance capability and promote tumoral growth.

Furthermore, a meta-analysis including 122 studies demonstrated that marriage and living with another person could increase adherence modestly, and patients from cohesive families had higher adherence than those from families in conflicts (24). And many previous studies demonstrated higher compliance and adherence to the treatment was also associated with better survival outcomes (25, 26). In addition, the study from Makubate et al. (27) demonstrated that low adherence to tamoxifen or aromatase inhibitors for women with breast cancer increased the risk of death. And McCowan et al. (28) observed that women who had low adherence to tamoxifen were significantly at increased risk of death. In addition, it was demonstrated by Langenbach et al. (29) that marital status had a significant influence on the treatment delay of colorectal cancer, which influenced the survival of patients. Last but not least, Ou et al. (30) found that unmarried status was associated with low socioeconomic status, which was an independent poor prognostic factor for NSCLC survival.

Several limitations are worth mentioning in this study. First, the quality of marriage was not recorded in the SEER database, and therefore we cannot further evaluate its impact on NSCLC survival. Manne et al. (31) found that the closeness of the marital relationship had an influence on psychological adaptation to cancer. Second, changes in marital status after diagnosis were also unavailable in the SEER database. Third, other clinicopathological characteristics and therapeutic details, including R0, R1 or R2 resection, clinical or pathological stage, the size of ground-glass opacity and solid component, and the specific dosage of chemotherapy and radiotherapy were not recorded, which might confound the final results. Forth, other socioeconomic factors, including family income levels and medical insurance status, need further investigation.

## Conclusion

In conclusion, our study still fully indicates that married patients with NSCLC have better survival outcomes than unmarried patients with NSCLC. Therefore, enhanced surveillance with a more intense follow-up regimen should be implemented for unmarried patients to decrease the survival risk caused by marital status. In addition, efforts to provide unmarried patients with social and psychological support will increase positive emotions, which are associated with better survival in cancer by influencing the immune system. Furthermore, closer surveillance and more social and family support simultaneously improve patients’ adherence and compliance to the treatments, which eventually improve survival. This study demonstrated that unmarried NSCLC patients was associated with significantly worse OS and CSS compared to married NSCLC patients. Therefore, unmarried patients need not only closer surveillance, but also more social and family support, which may improve patients’ adherence and compliance, and eventually improve the survival.

## Data availability statement

According to the regulation of the SEER database, we obtained permission to access the research data, and the reference number was 14683-Nov2019.

## Ethics statement

Ethical review and approval was not required for the study on human participants in accordance with the local legislation and institutional requirements. Written informed consent from the [patients/ participants OR patients/participants legal guardian/next of kin] was not required to participate in this study in accordance with the national legislation and the institutional requirements.

## Author contributions

LZ, DZ, and RZ conceived the idea and designed the research. DZ and RZ contributed to data collection and analysis. DZ, RZ, LY, ZH, YL, YW, GW, GG, and LZ interpreted the outcomes and wrote the paper together. All authors contributed to the article and approved the submitted version.

## Conflict of interest

The authors declare that the research was conducted in the absence of any commercial or financial relationships that could be construed as a potential conflict of interest.

The reviewer YX declared a shared parent affiliation with the authors to the handling editor at the time of review.

## Publisher’s note

All claims expressed in this article are solely those of the authors and do not necessarily represent those of their affiliated organizations, or those of the publisher, the editors and the reviewers. Any product that may be evaluated in this article, or claim that may be made by its manufacturer, is not guaranteed or endorsed by the publisher.

## Abbreviations

NSCLC: non-small cell lung cancer
SEER: surveillance, epidemiology and end results
PSM: propensity score matching
OS: overall survival
CSS: cancer specific survival
HR: hazard ratios
CI: confidence intervals
