# Supplementary figures and images for: The independent prognostic effect of marital status on non-small cell lung cancer patients: a population-based study
Source: Front Med (Lausanne). 2023 Jun 1;10:1136877. doi: 10.3389/fmed.2023.1136877 (PMC10267371; doi:10.3389/fmed.2023.1136877)

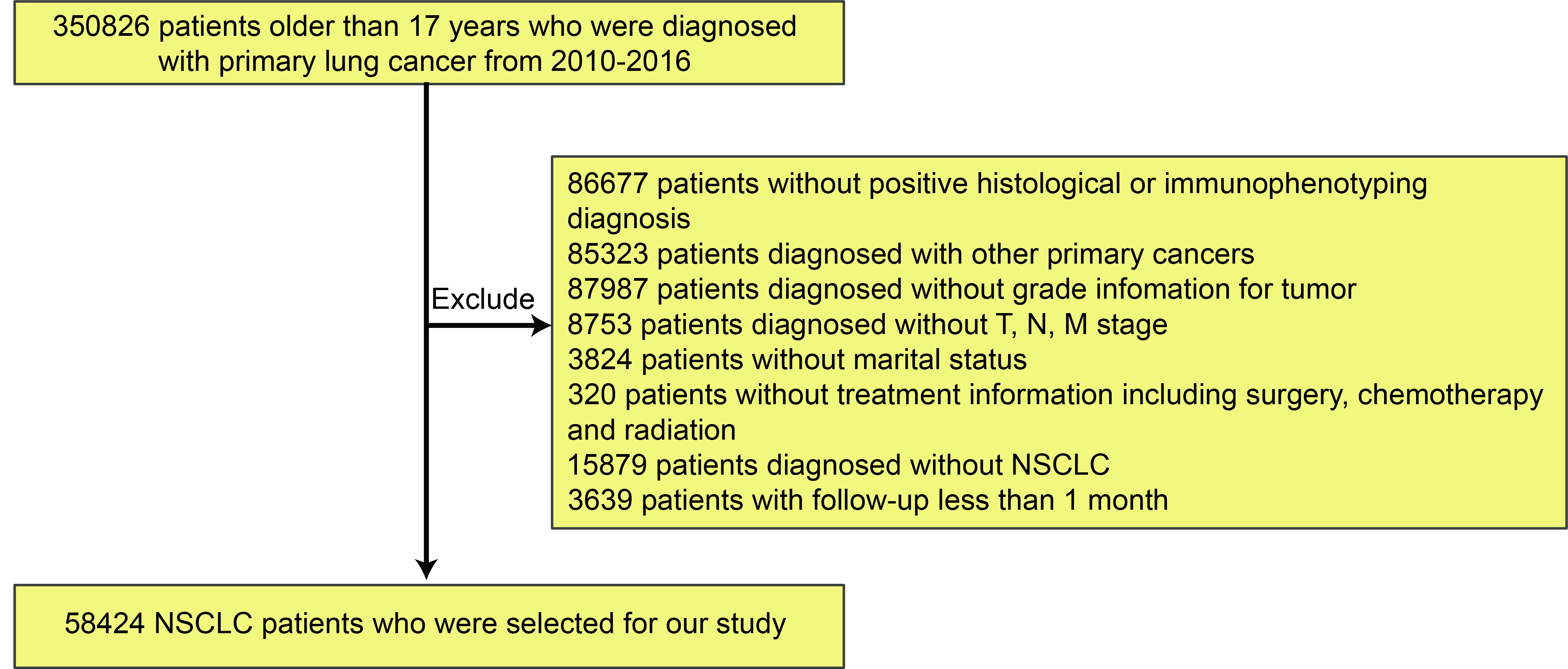

Supplement: Supplementary file 1 [file Image_1.TIF]

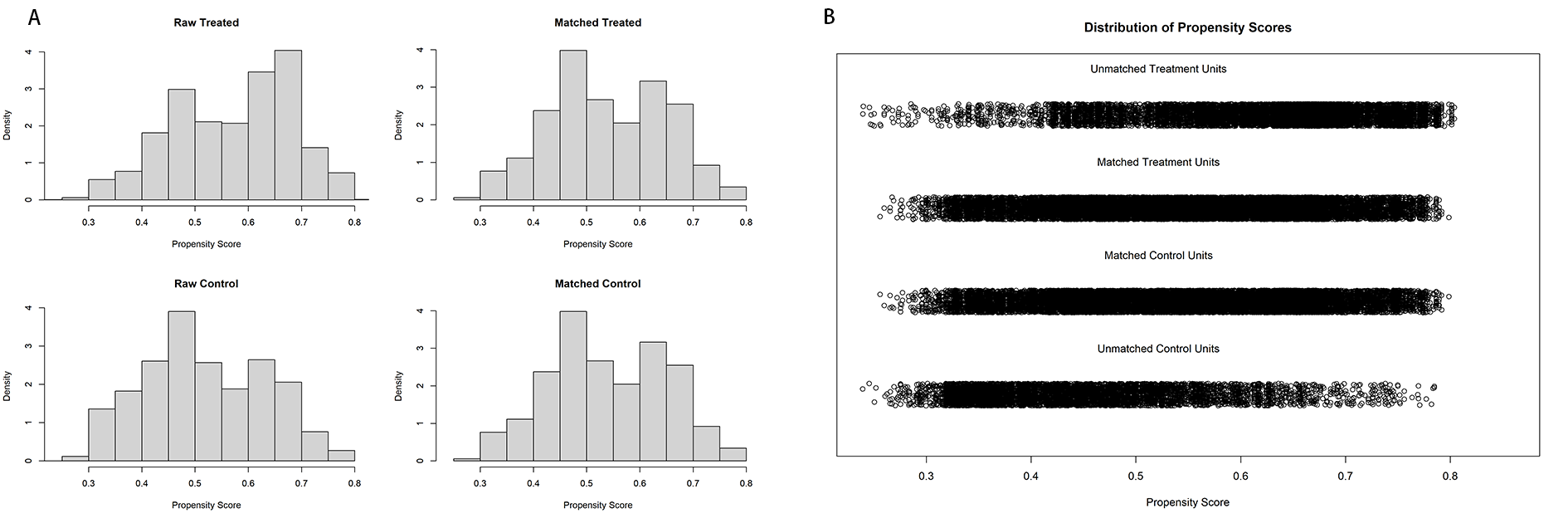

Supplement: Supplementary file 2 [file Image_2.TIF]
