# Supplementary material for: The independent prognostic effect of marital status on non-small cell lung cancer patients: a population-based study
Source: Front Med (Lausanne). 2023 Jun 1;10:1136877. doi: 10.3389/fmed.2023.1136877 (PMC10267371; doi:10.3389/fmed.2023.1136877)
Supplement: Supplementary file 4 [file Table_1.DOC]

Supplementary table 1. Clinicopathological characteristics of unmarried patients with NSCLC in SEER database before propensity score matching

| Variable | Single | Separated | Divorced | Widowed |
| --- | --- | --- | --- | --- |
|  | (n=8433,31.9%) | (n=694,2.6%) | (n=7640,28.9%) | (n=9632,36.5%) |
| Gender |  |  |  |  |
| Male | 4597(54.5%) | 363(52.3%) | 3478(45.5%) | 2539(26.4%) |
| Female | 3836(45.5%) | 331(47.7%) | 4162(54.5%) | 7093(73.6%) |
| Age |  |  |  |  |
| ≤65 | 5039(59.8%) | 424(61.1%) | 3707(48.5%) | 1340(13.9%) |
| ＞65 | 3394(40.2%) | 270(38.9%) | 3933(51.5%) | 8292(86.1%) |
| Race |  |  |  |  |
| White | 5783(68.6%) | 449(64.7%) | 6338(83.0%) | 7970(82.7%) |
| Black | 2150(25.5%) | 191(27.5%) | 973(12.7%) | 1024(10.6%) |
| Other | 500(5.9%) | 54(7.8%) | 329(4.3%) | 638(6.6%) |
| Histology |  |  |  |  |
| ADC | 5127(60.8%) | 411(59.2%) | 4474(58.6%) | 5499(57.1%) |
| SCC | 2931(34.8%) | 254(36.6%) | 2834(37.1%) | 3745(38.9%) |
| LCC | 205(2.4%) | 14(2.0%) | 182(2.4%) | 186(1.9%) |
| ASC | 170(2.0%) | 15(2.2%) | 150(2.0%) | 202(2.1%) |
| Grade |  |  |  |  |
| Well differentiated | 787(9.3%) | 65(9.4%) | 782(10.2%) | 1168(12.1%) |
| Moderately differentiated | 3201(38.0%) | 260(37.5%) | 3020(39.5%) | 3925(40.7%) |
| Poorly differentiated | 4283(50.8%) | 360(51.9%) | 3685(48.2%) | 4404(45.7%) |
| Undifferentiated | 162(1.9%) | 9(1.3%) | 153(2.0%) | 135(1.4%) |
| Stage |  |  |  |  |
| I | 2362(28.0%) | 211(30.4%) | 2462(32.2%) | 3448(35.8%) |
| II | 1060(12.6%) | 76(11.0%) | 1033(13.5%) | 1309(13.6%) |
| III | 1895(22.5%) | 170(24.5%) | 1751(22.9%) | 2036(21.1%) |
| IV | 3116(37.0%) | 237(34.1%) | 2394(31.3%) | 2839(29.5%) |
| T stage |  |  |  |  |
| 1 | 2011(23.8%) | 163(23.5%) | 2083(27.3%) | 2681(27.8%) |
| 2 | 2694(31.9%) | 229(33.0%) | 2456(32.1%) | 3381(35.1%) |
| 3 | 1831(21.7%) | 168(24.2%) | 1533(20.1%) | 1852(19.2%) |
| 4 | 1897(22.5%) | 134(19.3%) | 1568(20.5%) | 1718(17.8%) |
| N stage |  |  |  |  |
| 0 | 4020(47.7%) | 338(48.7%) | 3875(50.7%) | 5367(55.7%) |
| 1 | 901(10.7%) | 74(10.7%) | 799(10.5%) | 929(9.6%) |
| 2 | 2670(31.7%) | 212(30.5%) | 2257(29.5%) | 2610(27.1%) |
| 3 | 842(10.0%) | 70(10.1%) | 709(9.3%) | 726(7.5%) |
| M stage |  |  |  |  |
| 0 | 5317(63.0%) | 457(65.9%) | 5246(68.7%) | 6793(70.5%) |
| 1 | 3116(37.0%) | 237(34.1%) | 2394(31.3%) | 2839(29.5%) |
| Surgery of primary site |  |  |  |  |
| No | 5024(59.6%) | 395(56.9%) | 4130(54.1%) | 5659(58.8%) |
| Yes | 3409(40.4%) | 299(43.1%) | 3510(45.9%) | 3973(41.2%) |
| Intraoperative lymph node evaluation |  |  |  |  |
| No | 4961(58.8%) | 371(53.5%) | 4055(53.1%) | 5669(58.9%) |
| Yes | 3472(41.2%) | 323(46.5%) | 3585(46.9%) | 3963(41.1%) |
| Chemotherapy |  |  |  |  |
| No | 4556(54.0%) | 347(50.0%) | 4264(55.8%) | 6455(67.0%) |
| Yes | 3877(46.0%) | 347(50.0%) | 3376(44.2%) | 3177(33.0%) |
| Radiotherapy |  |  |  |  |
| No | 5072(60.1%) | 431(62.1%) | 4601(60.2%) | 6072(63.0%) |
| Yes | 3361(39.9%) | 263(37.9%) | 3039(39.8%) | 3560(37.0%) |

ADC Adenocarcinoma, SCC Squamous cell carcinoma, LCC Large Cell Carcinoma, ASC Adenosquamous carcinoma,
