# Supplementary material for: The independent prognostic effect of marital status on non-small cell lung cancer patients: a population-based study
Source: Front Med (Lausanne). 2023 Jun 1;10:1136877. doi: 10.3389/fmed.2023.1136877 (PMC10267371; doi:10.3389/fmed.2023.1136877)
Supplement: Supplementary file 5 [file Table_2.DOC]

Supplementary table 2. Clinicopathological characteristics of unmarried patients with NSCLC in SEER database after propensity score matching

| Variable | Single | Separated | Divorced | Widowed |
| --- | --- | --- | --- | --- |
|  | (n=6430,31.9%) | (n=547,2.7%) | (n=6161,30.6%) | (n=7010,34.8%) |
| Gender |  |  |  |  |
| Male | 3829(59.5%) | 313(57.2%) | 3104(50.4%) | 2356(33.6%) |
| Female | 2601(40.5%) | 234(42.8%) | 3057(49.6%) | 4654(66.4%) |
| Age |  |  |  |  |
| ≤65 | 3817(59.4%) | 334(61.1%) | 3106(50.4%) | 1062(15.1%) |
| ＞65 | 2613(40.6%) | 213(38.9%) | 3055(59.6%) | 5948(84.9%) |
| Race |  |  |  |  |
| White | 4980(77.4%) | 398(72.8%) | 5417(87.9%) | 6139(87.6%) |
| Black | 1040(16.2%) | 107(19.6%) | 469(7.6%) | 410(5.8%) |
| Other | 410(6.4%) | 42(7.7%) | 275(4.5%) | 469(6.6%) |
| Histology |  |  |  |  |
| ADC | 4080(63.5%) | 342(62.5%) | 3768(61.2%) | 4279(61.0%) |
| SCC | 2158(33.6%) | 190(34.7%) | 2182(35.4%) | 2531(36.1%) |
| LCC | 108(1.7%) | 8(1.5%) | 109(1.8%) | 90(1.3%) |
| ASC | 84(1.3%) | 7(1.3%) | 102(1.7%) | 110(1.6%) |
| Grade |  |  |  |  |
| Well differentiated | 608(9.5%) | 53(9.7%) | 653(10.3%) | 870(12.4%) |
| Moderately differentiated | 2521(39.2%) | 205(37.5%) | 2457(39.9%) | 2932(41.8%) |
| Poorly differentiated | 3221(50.1%) | 283(51.7%) | 2994(48.6%) | 3143(44.8%) |
| Undifferentiated | 80(1.2%) | 6(1.1%) | 75(1.2%) | 65(0.9%) |
| Stage |  |  |  |  |
| I | 1870(29.1%) | 171(31.3%) | 2026(32.9%) | 2616(37.3%) |
| II | 798(12.4%) | 59(10.8%) | 830(13.5%) | 913(13.0%) |
| III | 1375(21.4%) | 128(23.4%) | 1361(22.1%) | 1420(20.3%) |
| IV | 2387(37.1%) | 189(34.6%) | 1944(31.6%) | 2061(29.4%) |
| T stage |  |  |  |  |
| 1 | 1584(24.6%) | 134(24.5%) | 1709(27.7%) | 2044(29.2%) |
| 2 | 2074(32.3%) | 176(32.2%) | 1985(32.2%) | 2466(35.2%) |
| 3 | 1378(21.4%) | 136(24.9%) | 1245(20.2%) | 1312(18.7%) |
| 4 | 1394(21.7%) | 101(18.5%) | 1222(19.8%) | 1188(16.9%) |
| N stage |  |  |  |  |
| 0 | 3084(48.0%) | 277(50.6%) | 3121(50.7%) | 3939(56.2%) |
| 1 | 693(10.8%) | 55(10.1%) | 667(10.8%) | 677(9.7%) |
| 2 | 2001(31.1%) | 161(29.4%) | 1808(29.3%) | 1865(26.6%) |
| 3 | 652(10.1%) | 54(9.9%) | 565(9.2%) | 529(7.5%) |
| M stage |  |  |  |  |
| 0 | 4043(62.9%) | 358(65.4%) | 4217(68.4%) | 4949(70.6%) |
| 1 | 2387(37.1%) | 189(34.6%) | 1944(31.6%) | 2061(29.4%) |
| Surgery of primary site |  |  |  |  |
| No | 3623(56.3%) | 288(52.7%) | 3138(50.9%) | 3751(53.5%) |
| Yes | 2870(43.7%) | 259(47.3%) | 3023(49.1%) | 3259(46.5%) |
| Intraoperative lymph node evaluation |  |  |  |  |
| No | 3552(55.2%) | 270(49.4%) | 3063(49.7%) | 3769(53.8%) |
| Yes | 2878(44.8%) | 277(50.6%) | 3098(50.3%) | 3241(46.2%) |
| Chemotherapy |  |  |  |  |
| No | 3240(50.4%) | 257(47.0%) | 3260(52.9%) | 4447(63.4%) |
| Yes | 3190(49.6%) | 290(53.0%) | 2901(47.1%) | 2563(36.6%) |
| Radiotherapy |  |  |  |  |
| No | 3920(61.0%) | 350(64.0%) | 3766(61.1%) | 4534(64.7%) |
| Yes | 2510(39.0%) | 197(36.0%) | 2395(38.9%) | 2476(35.3%) |

ADC Adenocarcinoma, SCC Squamous cell carcinoma, LCC Large Cell Carcinoma, ASC Adenosquamous carcinoma,
